# Level and predictors of nurse caring behaviors among nurses serving in inpatient departments in public hospitals in Harari region, eastern Ethiopia

**DOI:** 10.1186/s12912-022-00856-8

**Published:** 2022-04-01

**Authors:** Haregeweyn Kibret, Barkot Tadesse, Adera Debella, Meron Degefa, Lemma Demissie Regassa

**Affiliations:** 1 Department of Nursing, School of Nursing and Midwifery, College of Health and Medical Sciences, Haramaya University, P.O.Box, 235, Harar, Ethiopia; 2 Department of Maternity and Neonatal Nursing, School of Nursing and Midwifery, College of Health and Medical Sciences, Haramaya University, PO Box 235, Harar, Ethiopia; 3 School of Public Health, College of Health and Medical Sciences, Haramaya University, PO Box, 235, Harar, Ethiopia

**Keywords:** Nursing practice, Caring behavior, Nurses, Inpatient services, Hospitals, Ethiopia

## Abstract

**Background:**

Nursing practice is centered on caring and nurses’ behaviour has an impact on the quality of patient care and it is predictive of patient satisfaction,however, many nurses, in reality, do not exhibit caring behavior when providing nursing care to clients. This study was aimed to assess the level and predictors of nurse caring behaviors among nurses serving in inpatient departments in public hospitals in Harari Region of Ethiopia from March 10 to April 10, 2021.

**Method:**

A cross-sectional study was conducted among 300 nurses providing inpatient service in public hospitals in the Harari region of eastern Ethiopia. All permanent nurses working in major inpatient services of two public hospitals, namely Jugal General Hospital (JGH) and Hiwot Fana Specialized University Hospital (HFSUH) were included. The English version of the CNPI-Nurse scale was used to determine the level of caring behavior. The association was reported using the crude and adjusted odds ratios along with the 95% confidence interval. The statistical significance of the association was declared at *p*-value < 0.05.

**Result:**

The caring behavior was classified as high and low based on the median score. According to this study only 51.67% (95% CI:45.97, 57.35%) of nurses had good caring behavior. The odds of having good caring behavior were 2.22 (AOR = 2.22, 95%CI: 1.20, 4.10) times higher among nurses working in good working environment compared to those who work in bad working environment. Nurses who were satisfied with their job had 2.79 (AOR: 2.79, 95%CI: 1.54, 5.08) times higher odds of good caring behavior than those who were not satisfied with their job. Similarly, nurses who had a lower workload had a 3.01 (AOR: 3.01, 95%CI: 1.70, 5.33) times higher probability of having good caring behavior from nurses compared to nurses who reported having a high workload.

**Conclusion:**

The level of nurses caring behavior is not satisfactory and it is influenced by working environment characteristics, job satisfaction and workload. Therefore it necessary to creat conducive working environment, provide adequate time and resources inorder to improve the level of nurses caring behaviour.

## Introduction

Nursing practice is centered on caring behavior that has an impact on patient care quality and is predictive of patient satisfaction [1–3]. Nursing care behaviour is an act, behaviour, and mannerism enacted by professional nurses that convey concern, safety, and attention to the patient. Caring behaviour has a critical part in tying nurse interactions to the client’s experiences [4, 5]. Constant evaluations of nurses’ caring conduct and patient satisfaction help to improve nursing quality [6].

Nursing is a caring profession that is defined by holistically treating all people, with a focus on meeting the patient’s basic needs, as well as their values and experiences. Any action that disregards patients as individuals or their worth and experience should be seen as uncaring and unethical behaviour [3, 7].

Caring behaviours might be influenced by different factors including workload, lack of time, staffing issues, shift work, and lack of self-care. Although it has been reported that nurses might care too much and get over-involved with their patients to the extent of visiting them on off days or buying them gifts Many nurses, in reality, do not exhibit caring behavior when providing nursing care to clients [8–10].

Clarifying the factors that may influence nurses’ perceptions of caring behaviors remains a highly important issue for the international nursing community [11]. In a prior study, nurses ranked psychological components of care as significant caring behaviors, but instrumental caring behaviors such as ‘know how to give shots, IV,’ and ‘know how to handle equipment’ did not make the list of the most important caring behaviors [12]. Even if there are differences between institutions, most health care providers focus on treating diseases rather than treating the person as a whole person; nonetheless, in nursing practice, the holistic approach is the most important part of nursing [13]. Nurses have a long period of interaction with patients and their families; caring behaviors are important when performing diagnostic and therapeutic interventions; however, there is no proper implementation of caring behaviors during hospitalization. This in turn creates patient dissatisfaction and poor nurse care delivery, resulting in a poor prognosis [6, 14]. Identifying the elements that influence nurses’ caring behavior is crucial to enhancing patient care quality [15]**,** Hence we are aiming to assess the level and determinants of nurse caring behaviors among nurses serving in inpatient departments in public hospitals in Harari Region of Ethiopia from March 10 to April 10, 2021.

## Methods and materials

### Study setting, period, and design

The institution-based cross-sectional study was conducted in public hospitals of the Harari region, which is found in eastern Ethiopia from March 10 to April 10, 2021. There are ten national regional states and two Administrative states (Addis Ababa City administration and Dire Dawa city council) Harari Region is among the ten regional states found in Ethiopia. Administratively, Harari Regional State is divided into six urban and three rural Woredas [16]. There are 2 public hospitals (HFSUH and JH), 1 military and 1 private hospitals,8 health centers (4 urban and 4 rural), 19 health posts, and ten private clinics in the region. Among them, HFSUH and JH provide multidimensional care to patients who need highly qualified/ specialized health care services. Hiwot Fana specialized university hospital (HFSUH) is teaching hospital for Haramaya University with a total of 161 beds and having medical, surgical, gynecology, pediatrics, and psychiatric wards. Jugal Hospital is a regional referral hospital in the Harari region that consists of medical, surgical, and gynecological services with 95 beds. Upto data collection date there were 240 and 116 nurses serving in inpatient service of HFSUH and JH respectively.

### Population and eligibility

Nurses providing inpatient services in public hospitals in the Harari region of eastern Ethiopia during the data collection period were included. Care providers on vacation and on special training were excluded from this study.

### Sample size determination and sampling procedure

To determine the sample size for this study, outcome variables and various factors significantly associated with the outcome variable were considered. Accordingly, for each specific objective the sample size is calculated separately and the larger sample size was taken to be used for this study. For the first objective, the magnitude of caring behavior of the the high level of nurses caring behaviour (*p* = 31.9%) is obtained from a previous study conducted in the Jimma referral hospital, the hospital in South-East Ethiopia [5]. Hence the calculated sample size was 334 the sample size for the second specific objective is determined by considering factors that are significantly associated with the outcome variable, the two-sided confidence level of 95%, the margin of error of 5%, power of 80% and the ratio of exposed to unexposed 1:1 using STAT CALC of Epi Info Version 7. Finally, the required sample size for this particular study is decided by taking the maximum from the sample size calculated was **334**. With a 5% nonresponse rate, the final sample size was 351, however, the total number of nurses providing inpatient service was 351. Hence, all nurses providing inpatient service of both Public hospitals (JGH and HFSUH) were included.

### Data collection methods

The socio-demographic data questionnaire was developed by reviewing previously published similar articles and other relevant works of literature [11, 17, 18]. The English version of the CNPI-Nurse scale with the reliability of cronach alpha 0.94 [19] was used to determine the level of caring behaviour. The CNPI tools contain 23 items with four main dimensions: clinical care, relational care, Humanistic care and comfort care components and the items were answered on a five-point Likert scale with the response options ranging from 1 (Rarely) to 5 (always). Four BSc nurse data collectors and two MSc supervisors were involved in the data collection process.

### Study variables and definitions

The level of nurses caring behavior was the outcome variable, job satisfaction, work environment characteristics and sociodemographic factors including age, sex, residedence, marital status, work experience, level of education, religion and working unit were considered as predictors of nurses caring behavior.

Caring behavior: is measured by The Short Scale comprises 23 items, reflecting four caring domains: Humanistic Care (four items), Relational Care (seven), Clinical Care (nine), and Comforting Care (three). Then caring behavior was classified as high and low based on the median score [19].

Clinical care is the component of caring behavior of the nurses that measures the aspects of clinical skills of nurses with nine items [19]. Relational care reflect the philosophical aspect of caring that measures the relationship with patients using seven items [19]. Humanistic care emphasizes the personal worth of the individual, the centrality values, and the active nature of nurses with their patients. It is measured by four items [19]. Comforting care: Items are more representative of the hidden work of nursing related to the comfort of patients. It is measured by three items (20). Job satisfaction: Three subscales of the operationalized nurse job satisfaction: (1) professional (5-items), (2) personal satisfaction (5-items) and (3) satisfaction with pay and prospect (4-items). The MMSS is nurse-specific scale developed using the theoretical work of Maslow. It uses a 5-point Likert scale from 1 ‘very dissatisfied’ to 5 ‘very satisfied’ [2]. Professional satisfaction: measures the satisfaction of nurses related to their work and the relationship with colleagues using five items [2]. Personal satisfaction: measures the perception of nurses related to their skills and challenges in the workplace using five items [2]. Satisfaction with pay and prospect: measures job-related praises and recognition from the organization for their achievement using four items [2].

### Data quality control

Since English is the media of instruction in higher institutions in Ethiopia English version of self-administered questionnaires was used and Pretests was conducted on 5% of the sample size in Haramaya hospital which is out of the study area and the necessary corrections was made based on the results of the pre-test. The training was given for data collectors and supervisors for 1 day on data collection tools. Data collectors gave a brief introductory orientation to study participants on the purposes of the study. The investigators and the supervisors monitored the data collection process, and check for the completeness and logical consistencies of the collected data and give appropriate feedback accordingly. Intensive supervision was provided by the supervisors and the principal investigator.

### Data processing and analysis

The collected data was checked for its completeness and cleaned before entry into the computer. The questionnaire was then coded and the data was entered into Epi data version 3.1 by two data clerks who were recruited. The change in the nursing care behavior score of the caring behavior of nurses was estimated using the facto score. Furthermore, we fitted linear regression to identify the association of the working environment and workload with the change in the caring behavior score of nurses. Three models were fitted to assess the determinants of caring behaviour of nurses. The first two models were fitted to analyze the change of caring behavior score with workplace interaction and job satisfaction using linear regression. Finally determinants of Nurses caring behaviour was identified by using logistic regression, after the level of caring behavour was dichotomized using median score. The association was reported using the crude odds ratio (COR) and adjusted odds ratio (AOR) along with the 95% confidence interval (CI). Using stepwise methods, we constructed a variety of regression models from the set of variables including sociodemographic factors, job satisfaction, work environment conditions, and work expereriance. The best-fit model was selected using information criteria (AIC and BIC). Model fitness and sensitivity analysis were done by appropriate methods (chi-square or residual fitness). Finally, the statistical significance was declared at a *p*-value < 0.05.

## Results

### Socio demographic characteristics

Of the 351 expected participants, 300 of them participated in this study, making the response rate 85%. The left 35 (10%) refused to participate and 16 (5%) did not answer all components of questiones that define caring behaviour. The mean (± standard deviation (SD)) age of the nurses was 28.07 (±7.09) years. Almost half of the respondents (*n* = 147, 49%) were in the age group 25–34 years. More than half (*n* = 169, 56.33%) were women, about (*n* = 141, 47%) of the participants were Orthodox. The majority (*n* = 239, 79.67%) of the respondents have a BSc in nursing. Regarding the working unit of the respondent (*n* = 160, 53.33%) was working on the medical ward. Majority (*n* = 212, 70.67%) of them had 5 years and below work experience (Table 1).

**Table 1:** Sociodemographic characteristics of nurses working in public hospitals in the Harari region of eastern Ethiopia

Variables	Frequency	Percent
Working hospital		
HFSUH	171	57
Jugal hospital	129	43
Age in years		
< =24	109	36.33
25–34	147	49
> = 35	44	14.67
Sex		
Female	169	56.33
Male	131	43.67
Religion		
Orthodox	141	47
Muslim	120	40
Protestant	37	12.33
Other	2	0.67
Marital status		
Married	131	43.67
Single	169	56.33
Educational status		
Diploma	48	16
BSc	239	79.67
MSc	13	4.33
Working unit		
Medical	160	53.33
Surgical	98	32.67
Pediatrics	26	8.67
Gyne_obse	16	5.33
Work experience		
< = 5 yr	212	70.67
> = 6 yr	88	29.33

Other = wakafataa, HFSUH=Hiwotfana specialized university Hospital, Gyne_Obse = gynecology and obsetrics, yr = year

Among the respondents, half 150(50%) of the nurses were satisfied with their job. From the subscales of job satisfaction 181 (60.33) of them had “professional satisfaction’, 158 (52.67%) of nurses had “personal satisfaction” and 143(47.67%) of them were satisfied with the “pay-prospect. Moreover, more than half 157(52.33%) of the nurses reported that they had good working environment and 173(58%) of reported that they had workload.

### The level of caring behavior of nurses

The median (±IQR) of the overall scor of the caring behavior of the nurses was 84.04 (±0.95) and 51.67% of the nurses had a high caring behaviour. The median (±IQR) score (IQR) for each component was 34.80 (±0.42) for clinical care, 22.17 (±0.30) for relational care, 15.07(±0.21) for humanistic care, and 11.99 (±0.16) for comforting care.

**Fig. 1:**
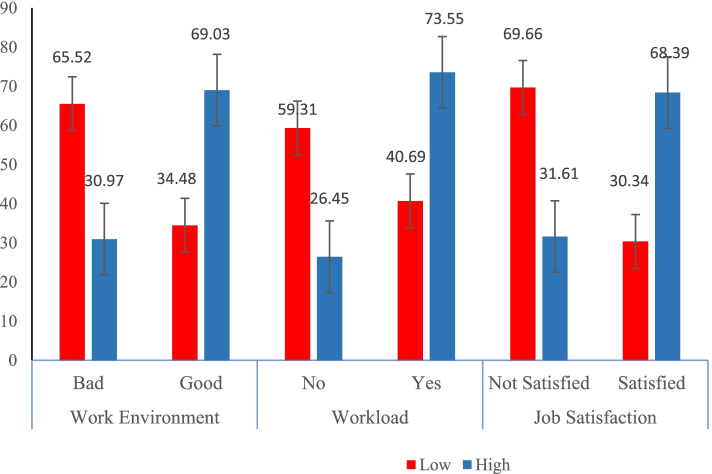
magnitude of nurses caring behaviour stratifying by workload, working environment and job satisfaction of nurses. Legends: Red color key for low proportion, and blue color key for high proportion

Regarding the subscales of work environment characteristics, nearly half (51%) of them reported there is adequate staffing and resource, while 60.7% of them reported good nurse-doctor relationship and 52.7% of them reported good nurse management (Fig. 1). Figure 1 summarizes magnitude of nurses caring behaviour stratifying by workload, working environment and job satisfaction of nurses.

### Predictors of nurses caring behaviour

Three models were fitted to determine the association of the work environment and job satisfaction. In all models, both work environment and job satisfaction were significantly associated with caring behavior of nurses. In the first model, where sociodemographic factors are controlled, nurse management is negatively associated with caring behavior and staffing resources are positively associated with caring behavior. As the score of nurse management increased by one scale, the score of nurse caring behavior decreased by 60% (β = 0.60, 95%CI: 0.26, 0.95). In contrast, caring behavior was increased on a scale of 1.23 (β = 1.23, 95%CI: 0.75, 1.71), as the score for staffing resource was increased on a scale (Table 2).

**Table 2:** Association of caring behavior score of nurses with the status of relationship with other coworkers in the workplace (model 1)

Work Environment characteristics	Β (95%CI)	***p***-value
**Nurse managements**	**0.60 (0.26, 0.95)**	**0.001**
Nurse doctor	0.57 (−0.09, 1.24)	0.091
**Staffing resource**	**1.23 (0.75, 1.71)**	**< 0.001**

Controlled for; sex, age, educational status, work unit, marital status, working experience, workload

Regarding the job satisfaction, the personal component was significantly associated with higher score of nurses caring behavior (β = 1.65, 95%CI: 1.15, 2.15). On the other hand, the association of caring behavior of nurses with professional and pay and prospect components was not significant (Table 3).

**Table 3:** Association of job satisfaction with caring behavior among nurses

Satisfaction with	Β (95%CI)	***p***-value
Professional	0.54 (− 0.12, 1.20)	0.106
**Personal**	**1.65 (1.15, 2.15)**	**0.011**
Pay and prospect	−0.06 (− 0.52, 0.41)	0.809

Controlled for; sex, age, educational status, work unit, marital status, work experience, workload, work environment characteristics

The final model was fitted after the score of nurses caring behavior, job satisfaction, and working environment characteristics were categorized into binary using factor analysis. In the final multiple logistic regression nurses working environment, job satisfaction and workload were significantly associated with nurse’s caring behavior.

The odds of having good caring behavior were 2.22 (AOR = 2.22, 95%CI: 1.20, 4.10) times higher among nurses working in good working environment compared to those who work in bad working environment. Similarly, those who satisfied with their job had 2.79 (AOR: 2.79, 95%CI: 1.54, 5.08) times higher odds of good caring behavior than those who did not satisfied with their job. On the other hand, nurses who had a lower workload had 3.01 (AOR:3.01, 95%CI:1.70, 5.33) times higher odds of having good nurses’ caring behavior compared to nurses who reported having a high workload (Table 4).

**Table 4:** Predictors of the caring behavior of nurses in public hospitals in Harari region, Eastern Ethiopia

Variable	AOR (95%CI)	*p*-value
Working environment		
Good	2.22 (1.20, 4.10)	0.011
Bad	1	
Job satisfaction		
Satisfied	2.79 (1.54, 5.08)	0.001
Not satisfied	1	
Workload		
No	3.01 (1.70, 5.33)	< 0.001
Yes	1	
Sex		
Male	0.91 (0.53, 1.58)	0.75
Female	1	
Age categories (years)		
≤ 24	1	
25–34	1.67 (0.88, 3.15)	0.115
≥ 35	0.89 (0.29, 2.75)	0.835
Experience (years)		
< 6	1	
≥ 6	0.78 (0.35, 1.71)	0.531
Working Unit		
Medical	0.99 (0.31, 3.21)	0.992
Pediatrics	3.32 (0.76, 14.56)	0.112
Surgical	1.04 (0.31, 3.51)	0.949
Gynecological and obstetrics	1	
Educational status		
BSc.	1	
Diploma	0.87 (0.41, 1.87)	0.724
MSc.	0.81 (0.18, 3.67)	0.786
Marital status		
Not married	1.55 (0.82, 2.91)	0.178
Married	1	

*AOR* Adjusted Odds Ratio

## Discussion

According to this study, the proportion of nurses who had a high level of caring behavior was 51.67%. This finding is in contrast to the study done in Jimma where the proportion of nurses who had a high caring behavior was found to be lower [5]. This variation may occur due to the sample size they used was relatively smaller. Furthermore, they conducted the study at Jimma University specialized hospital only where the patient flow is expected to be higher, which can affect the caring behavior of the nurses. Additionally, the difference may occur due to variation in study tool used to measure caring behavior dimension.

The proportion high perception of caring behavior in this study was found to be lower than other study [20]. The difference may occur due to the previous study data was collected after implementation of a Relationship Centered Care Professional Practice Model. In this study, the majority of nurses had high humanistic care, which is the core and starting point of nursing and also an important indicator of quality care [21], followed relational care, clinical care and comforting care, respectively. These findings indicate that nurses, in our study area, are more concerned with empathy, respect for human dignity, altruism and other aspects of humanistic care and value humanity more than the technical-professional aspect of caring behavior. Our finding is in line with the study done in Philippines, china and Switzerland by using the same CNPI tool [22–24]. On the other hand, our finding is in contrast to the study done in Croatia where the highest subscale was the ‘need’ this variation may be due to the fact that nursing education in Croatia is highly based on Henderson’s theory of basic human needs [25]. According to [26] report, comforting care behavior was the highest from the caring behaviors assessed among the nurses.

This study finding outlined that Workload, job satisfaction and work environment characteristics were the factors significantly associated with the nurses caring behavior. The odds of having good caring behavior were 2.22 (AOR = 2.22, 95%CI: 1.20, 4.10) times higher among nurses working in good working environment compared to those who work in bad working environment, it is supported by [5, 14], satisfaction with nurse management was significantly associated with caring behavior which is one of the component of work environment characteristics and nurses who had bad relationship with doctors and conflict with supervisor were 4.56 times and 2.44 times more likely to have negative perception towards nurses caring behavior. Other study outlined that to reconstruct the nursing working environment to be healthier and boost the caring behavior of nurses, it is required to give supervision, contingent rewards, empowerment, and collaborative programs for nurses [1]. This study finding was also supported by another study which revealed that the variation in nurse care was explained by the satisfaction of nurse with social contact [2] .

According to this study, nurses who were satisfied with their job had 2.79 (AOR: 2.79, 95%CI: 1.54, 5.08) times higher odds of good caring behavior than those who were not satisfied with their job. This study is in line with the results of several studies, which reported that job satisfaction had a positive correlation with caring behavior of nurses and job satisfaction was significantly associated with caring behavior of nurses [1, 2, 5].

This study finding revealed that nurses who had lower workload were 3.01 (AOR: 3.01, 95%CI: 1.70, 5.33) times higher likelihood of having good nurses caring behavior as compared to nurses who reported they had high workload, and it is in agreement with the study used the Greek Version of the Caring Behaviors Inventory scale (CBI-GR) which has 4 correlated dimensions and the finding outlined that stress regarding [27]. Workload was negatively related to all CBI subscales [28]. Furthermore, support this study finding which outlined that externally imposed time pressures and mental effort, as well as job-level heavy workload demands and task-level interruptions, have a negative impact on patient and nurse outcomes.

This study is not without limitations. we did not assess facility and payment related factors. Some inpatient departments such as psychiatry were not included as the working environment is completely different from other wards. As a result, this study will not be generalized to all inpatient services in all settings.

## Conclusion

The level of nurses caring behavior is not satisfactory and it is influenced by working environment characteristics, job satisfaction and workload. Therefore it necessary to creat conducive working environment, provide adequate time and resources inorder to improve the level of nurses caring behaviour.

## Data Availability

The datasets generated and/or analyzed during this work are not publically available due to Haramaya University’s ethical protocol restricting data sharing, but they can be obtained from the corresponding author on reasonable request.

## Abbreviations

CNPI: Patient-clinical nurse interaction
PSI: Patient satisfaction instrument
HFSUH: Hiwot Fana Specialised University Hospital
JGH: Jugel General Hospital
IHRERC: Institutional Health Research Ethics Review Committe
